# Relationship of OCT-defined plaque characteristics with CCTA-derived coronary inflammation and CMR-derived global coronary flow reserve in patients with acute coronary syndrome

**DOI:** 10.1371/journal.pone.0286196

**Published:** 2023-05-25

**Authors:** Tomoyo Sugiyama, Yoshihisa Kanaji, Masahiro Hoshino, Masahiro Hada, Toru Misawa, Tatsuhiro Nagamine, Yun Teng, Kai Nogami, Hiroki Ueno, Kazuki Matsuda, Kodai Sayama, Eisuke Usui, Tadashi Murai, Tetsumin Lee, Taishi Yonetsu, Tetsuo Sasano, Tsunekazu Kakuta

**Affiliations:** 1 Division of Cardiovascular Medicine, Tsuchiura Kyodo General Hospital, Ibaraki, Japan; 2 Department of Cardiovascular Medicine, Tokyo Medical and Dental University, Tokyo, Japan; Universitatsklinikum Wurzburg, GERMANY

## Abstract

**Background:**

The relationship of layered plaque detected by optical coherence tomography (OCT) with coronary inflammation and coronary flow reserve (CFR) remains elusive. We aimed to investigate the association of OCT-defined layered plaque with pericoronary adipose tissue (PCAT) inflammation assessed by coronary computed tomography angiography (CCTA) and global (G)-CFR assessed by cardiac magnetic resonance imaging (CMR) in patients with acute coronary syndrome (ACS).

**Methods:**

We retrospectively investigated 88 patients with first ACS who underwent preprocedural CCTA, OCT imaging of the culprit lesion prior to primary/urgent percutaneous coronary intervention (PCI), and postprocedural CMR. All patients were divided into two groups according to the presence and absence of OCT-defined layered plaque at the culprit lesion. Coronary inflammation was assessed by the mean value of PCAT attenuation (−190 to −30 HU) of the three major coronary vessels. G-CFR was obtained by quantifying absolute coronary sinus flow at rest and during maximum hyperemia. CCTA and CMR findings were compared between the groups.

**Results:**

In a total of 88 patients, layered plaque was detected in 51 patients (58.0%). The patients with layered plaque had higher three-vessel-PCAT attenuation value (-68.58 ± 6.41 vs. -71.60 ± 5.21 HU, P = 0.021) and culprit vessel-PCAT attenuation value (-67.69 ± 7.76 vs. -72.07 ± 6.57 HU, P = 0.007) than those with non-layered plaque. The patients with layered plaque had lower G-CFR value (median, 2.26 [interquartile range, 1.78, 2.89] vs. 3.06 [2.41, 3.90], P = 0.003) than those with non-layered plaque.

**Conclusions:**

The presence of OCT-defined layered plaque at the culprit lesion was associated with high PCAT attenuation and low G-CFR after primary/urgent PCI in patients with ACS. OCT assessment of culprit plaque morphology and detection of layered plaque may help identify increased pericoronary inflammation and impaired CFR, potentially providing the risk stratification in patients with ACS and residual microvascular dysfunction after PCI.

## Introduction

Pathology studies have suggested that subclinical episodes of coronary thrombosis caused by plaque rupture or erosion and subsequent healing lead to plaque progression and myocardial injury [1–3]. Histologically-defined healed plaque is characterized by a layered structure on in vivo optical coherence tomography (OCT) imaging [4]. Thus, the presence of layered plaque is suggestive of recurrent thrombotic events and myocardial injury. However, the relationship of OCT-defined layered plaque with coronary inflammation and coronary flow reserve (CFR) remains unclear in patients with acute coronary syndromes (ACS).

Recent studies have demonstrated the feasibility and clinical implication of coronary computed tomography angiography (CCTA)-derived pericoronary adipose tissue attenuation (PCAT) for non-invasive assessment of coronary inflammation and risk stratification [5, 6]. In the present study, we hypothesized that the presence of OCT-defined layered plaque may be associated with increased PCAT inflammation assessed by CCTA and decreased global (G)-CFR assessed by cardiac magnetic resonance imaging (CMR) in patients with ACS.

## Methods

### Study population

**Fig 1:**
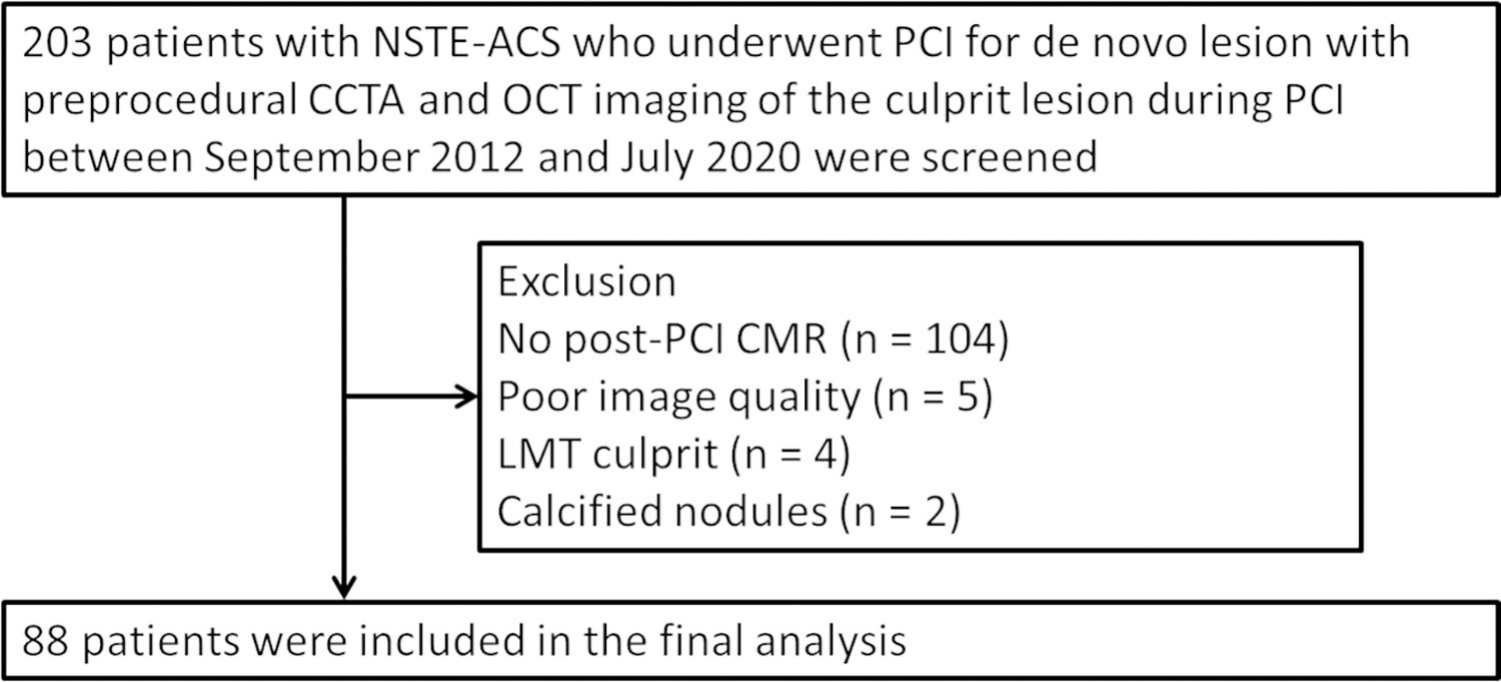
Study cohort.

**Fig 2:**
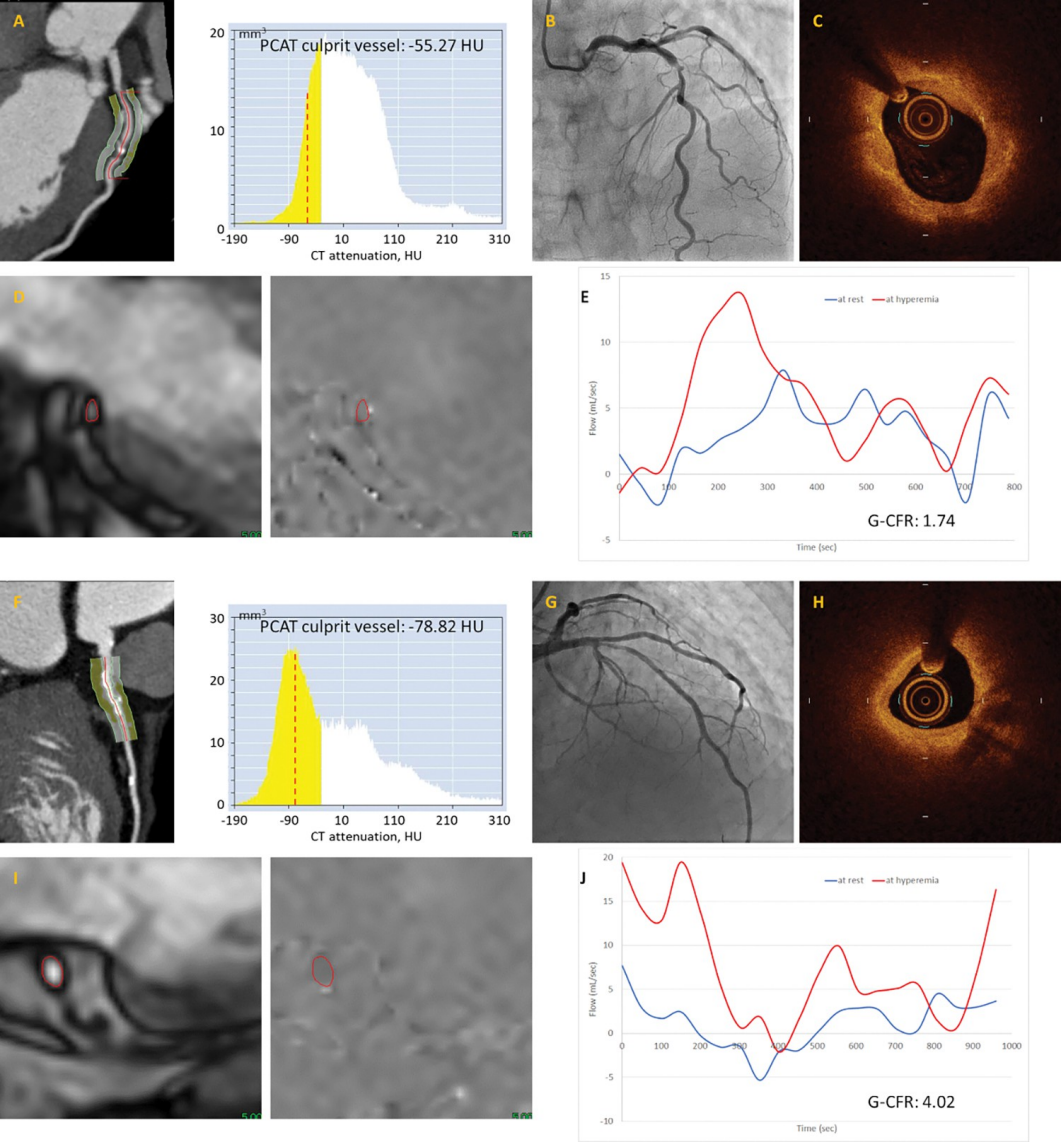
Representative images of patients with and without layered plaque. (Upper panel) A case of non-ST-segment-elevation acute coronary syndrome (NSTE-ACS) with layered plaque. (A) Coronary computed tomography angiography (CCTA) revealed that the pericoronary adipose tissue (PCAT) attenuation value of the left anterior descending artery (LAD) and that of the three-vessel-PCAT were -55.27 HU and -60.14 HU, respectively. (B) Coronary angiography revealed a stenosis in the proximal LAD. (C) Optical coherence tomography (OCT) revealed the presence of layered plaque. (D, E) Phase contrast (PC) cine-cardiac magnetic resonance imaging (CMR) of the coronary sinus measurement revealed that the global coronary flow reserve (G-CFR) was 1.74. (Lower panel) A case of NSTE-ACS with non-layered plaque. (F) CCTA revealed that the PCAT attenuation value of the LAD and that of the three-vessel-PCAT were -78.82 HU and -75.12 HU, respectively. (G) Coronary angiography revealed a stenosis in the proximal LAD. (H) OCT revealed the absence of layered plaque. (I, J) PC-CMR revealed that the G-CFR was 4.02.

This retrospective analysis included patients from the institutional OCT registry database enrolled between September 2012 and July 2020 at Tsuchiura Kyodo General Hospital. In the present study, patients with non-ST-segment-elevation (NSTE)-ACS who underwent preprocedural CCTA, OCT imaging of the culprit lesion prior to primary/urgent percutaneous coronary intervention (PCI) and postprocedural CMR within 30 days after the index PCI were retrospectively enrolled. Patients presenting with NSTE-ACS and stable hemodynamics underwent CCTA within a few hours after arrival in the emergency department to rule out patients with no significant epicardial stenosis [7] and to obtain diagnostic information regarding culprit lesion location, atherosclerotic burden and preprocedural planning of revascularization, using a relatively low dose of radiation and contrast. Patients with a history of known MI, PCI, and/or coronary artery bypass grafting were excluded. Patients with myocardial infarction with non-obstructive coronary arteries, spontaneous coronary artery dissection, and takotsubo myocardiopathy were also excluded. In patients with multivessel disease, when significant non-infarct-related artery stenoses were considered as candidates for revascularization by physiological assessment, ad-hoc procedure at the time of the index primary/urgent PCI or a planned staged procedure during the index hospitalization was performed. Postprocedural CMR was performed after non-infarct-related lesion revascularization. Thus, a total of 88 patients with first NSTE-ACS who underwent PCI for de novo lesion with preprocedural CCTA, OCT imaging of the culprit lesion, and postprocedural CMR were included in the final analysis (Fig 1). All patients were divided into two groups according to the OCT-defined culprit plaque characteristics: layered vs. non-layered plaque. Patients’ clinical characteristics, CCTA and CMR findings, angiographic and OCT findings of the target lesion were compared between these two groups. Representative images are shown in Fig 2.

### Ethical approval

This study was conducted in compliance with the Declaration of Helsinki for investigation in human beings. The study protocol was reviewed and approved by the institutional ethics committee on human research of Tsuchiura Kyodo General Hospital (approval number: 2022FY71). All patients provided written informed consent for enrollment in the institutional OCT registry database of Tsuchiura Kyodo General Hospital for potential future investigations. All patient data and procedural details were obtained from their medical records and the institutional OCT registry database.

### CCTA image acquisition

CT examinations were performed using a 320-slice CT scanner (Aquilion ONE; Canon Medical Systems Corporation, Otawara, Tochigi, Japan), as previously described [8, 9]. In brief, CCTA images were acquired with the scan protocol as follows: tube voltage of 120 kVp, tube current of 50 to 750 mA, gantry rotation speed of 350 ms per rotation, field matrix of 512 × 512, and scan slice thickness of 0.5 mm. CCTA images were reconstructed at a window centered at 75% of the R-R interval to coincide with left ventricular (LV) diastasis.

### PCAT analysis

PCAT attenuation analysis was performed using semiautomated software (Aquarius iNtuition Edition version 4.4.13.P3; TeraRecon Inc., Foster City, CA, USA), as previously described [5, 8]. PCAT was defined as all voxels with CT attenuation between −190 and −30 HU located within a radial distance from the outer coronary wall equal to the diameter of the vessel [5, 8]. PCAT attenuation was defined as the average CT attenuation of adipose tissue within the defined region of interest [10]. PCAT attenuation was measured in the proximal 40-mm segment of the left anterior descending coronary artery (LAD) and left circumflex coronary artery (LCx) and the proximal 10- to 50-mm segment of the right coronary artery (RCA) [5]. Three-vessel-PCAT attenuation defined as the mean PCAT attenuation of the three major coronary arteries was also calculated. PCAT analysis was performed by investigators who were blinded to clinical, OCT, and CMR data.

### Coronary catheterization and PCI procedure

Each patient underwent selective coronary angiography via the radial artery with a 5-French or 6-French system. All patients underwent PCI with coronary drug-eluting stent implantation. The stent type was selected at the operator’s discretion, and the PCI strategy was determined by the interventionist. Quantitative coronary angiography (QCA) and OCT findings were used to help determine the appropriate stent size and obtain optimal stent expansion.

### QCA analysis

Conventional QCA analysis was performed using an offline analysis software (QAngio XA 7.3.74.0; Medis medical imaging systems bv, Leiden, The Netherlands). The minimal lumen diameter (MLD), reference vessel diameter, diameter stenosis (DS), and lesion length of the target lesions were measured [11].

### OCT image acquisition and analysis

OCT examination was performed using frequency-domain OCT systems: Abbott’s OCT (Ilumien Optis, Abbott Vascular, Santa Clara, CA, USA) or Terumo’s optical frequency-domain imaging system (Lunawave, Terumo Corporation, Tokyo, Japan), as previously described [12, 13]. OCT pullback was performed automatically by the dedicated device during the injection of flushing agents, either contrast medium or low-molecular-weight dextran with Ringer’s lactate solution, at a flow rate of 3–4 mL/s via the guiding catheter using an automated power injector pump. All OCT images were analyzed using an offline review workstation by two independent investigators who were blinded to clinical, CCTA, and CMR data. Any discordance was resolved by consensus with a third reviewer. Cross-sectional OCT images were analyzed at 1-mm intervals. Minimal lumen area (MLA) was defined as the smallest lumen area within the length of the plaque [14]. Lipid was defined as a signal-poor region with a poorly defined or diffuse border and the degree of lipid arc was measured in lipid plaques [14]. Fibrous cap thickness (FCT) overlying a lipid plaque was measured three times at its thinnest part and the average value was calculated [15]. Lipid-rich plaque was defined as a plaque with a maximal lipid arc >90 degrees [15]. Thin-cap fibroatheroma (TCFA) was defined as a plaque with maximal lipid arc >90 degrees and thinnest FCT ≤80 μm [15]. Macrophage accumulation was defined as the presence of highly backscattering focal regions within the fibrous cap [16]. Microvessels were defined as the presence of signal-poor structures with vesicular or tubular shape [16]. Cholesterol crystals were identified as thin and linear regions of high signal intensity with high backscattering within a plaque [16]. Calcification was defined as signal-poor or heterogeneous regions with sharply delineated borders [16]. Thrombus was defined as an irregular mass with minimal diameter >250 μm adherent to the vessel wall or floating within the lumen [16]. Plaque rupture was defined as fibrous cap discontinuity with cavity formation [16]. Layered plaque was identified by the presence of one or more signal-rich layers of different optical density and a clear demarcation from underlying plaque components [4, 17]. In patients with multiple stenoses, the culprit lesion was identified as the lesion having the most severe stenosis and/or with evidence of recent plaque disruption, including thrombus. Intraobserver and interobserver agreement in the diagnosis of layered plaque assessment was excellent (kappa = 0.907 and 0.815, respectively).

### CMR image acquisition and analysis

CMR image acquisition was performed using a 1.5-Tesla scanner (Achieva, Philips Medical Systems, Best, The Netherlands) with 32-channel cardiac coils within 30 days after the index PCI, as previously described [18]. In brief, cine-CMR images were acquired with the scan protocol as follows: repetition time, 4.1 ms; echo time, 1.4 ms; slice thickness, 6 mm; flip angle, 55 degrees; field of view, 350 × 350 mm^2^; matrix size, 128 × 128; and number of 20 phases per cardiac cycle, 20. LV mass and volumes were calculated according to the Simpson’s rule. Late gadolinium enhancement (LGE) images were acquired 15 min after the injection of gadolinium contrast (0.10 mmol/kg) using inversion recovery prepared gradient echo sequences as follows: repetition time, 3.8 ms; echo time, 1.28 ms; slice thickness, 8 mm; flip angle, 15 degrees; field of view, 350 × 350 mm^2^; acquisition matrix, 200 × 175; and number of phases per cardiac cycle, 20. All CMR images were analyzed using a dedicated workstation (Virtual Place, AZE Ltd., Tokyo, Japan) by two independent investigators who were blinded to clinical, angiographic, and OCT data. The infarcted myocardium was quantified on the LGE images as myocardium with a signal intensity exceeding the mean signal intensity of the remote myocardium by ˃5 standard deviation (SD) by using a semi-automatic algorithm. The plane for flow measurement by phase contrast (PC) cine-CMR was positioned perpendicular to the coronary sinus (CS) at ≈1 to 2 cm from the ostium [19]. Velocity-encoded images were acquired using retrospective electrocardiographic gating during 15-second breath holds, and the imaging parameters were as follows: repetition time, 7.3 ms; echo time, 4.4 ms; slice thickness, 6 mm; flip angle, 10 degrees; field of view, 250 × 250 mm^2^; acquisition matrix, 128 × 128; number of phases per cardiac cycle, 20; and encoding, 200 cm/s. PC-CMR of the CS measurements was performed during maximal hyperemia and at rest. Maximal stable hyperemia was induced by intravenous adenosine triphosphate (160 μg/kg/min through a central vein). Coronary sinus flow (CSF) quantitative analyses by PC-CMR were performed in a blinded fashion using proprietary software (View Forum, Philips Medical Systems, Best, The Netherlands) by two independent investigators who were blinded to clinical, angiographic, and OCT data. CS contour was traced on the magnitude images throughout the cardiac cycle. CSF was quantified by integrating the flow rates from each cardiac cycle and multiplying them by the mean heart rate during the acquisition period. CSF quantifications were performed during maximal hyperemia and at rest. The resting CSF value was corrected using rate pressure products as follows: rate pressure product (RPP) = systolic blood pressure (mmHg) × heart rate (bpm); corrected CSF (mL/min) = (CSF/RPP) × 10,000; and corrected CSF (mL/min per g) = corrected CSF/LV mass (g) [19, 20]. G-CFR was evaluated by corrected CSF reserve, which was calculated as CSF during maximal hyperemia divided by corrected CSF at rest [18]. The reproducibility of G-CFR measurements was satisfactory for interobserver (intraclass correlation coefficient, 0.91) and intraobserver (intraclass correlation coefficient, 0.89).

### Statistical analysis

All analyses were performed using SPSS Statistics 23.0 software (IBM Corporation, Armonk, NY, USA) and R for Windows 4.1.1 (The R Foundation, Vienna, Austria). Categorical data were expressed as absolute frequencies and percentages and compared using the chi-square test or Fisher’s exact test, as appropriate. Continuous variables were expressed as mean ± SD for normally distributed variables and as median (interquartile range [IQR]) for non-normally distributed variables, and compared using the Student’s t-test or Mann‒Whitney test, as appropriate. A P value <0.05 was considered statistically significant. Two prediction models were used to determine the incremental discriminatory and reclassification performance for identifying the predictors of G-CFR <2.0. Clinical model 1 as the reference model included established or historically reported significant factors associated with atherosclerotic burden (age, sex, hypertension, diabetes mellitus, current smoking, and culprit plaque rupture); and Clinical model 2 included a combination of Clinical model 1 and OCT-defined layered plaque. The discriminatory abilities of Clinical model 2 were assessed by the reclassification performance of each model using the relative integrated discrimination improvement (IDI) and category-free net reclassification improvement (NRI) values.

## Results

### Patient characteristics

In a total of 88 patients, the mean age was 64.5 years, and 74 patients (84.1%) were male. Layered plaque was detected in 51 patients (58.0%). Patients’ baseline clinical characteristics and laboratory findings of the study population are described in Table 1. Peak CK and CK-MB values were not significantly different between the patients with layered plaque and non-layered plaque.

**Table 1:** Baseline patient characteristics.

	All (n = 88)	Layered plaque (n = 51)	Non-layered plaque (n = 37)	P value
Age, years	64.5 ± 10.5	64.8 ± 10.2	63.7 ± 11.3	0.623
Sex				1.000
Male	74 (84.1)	43 (84.3)	31 (83.8)	
Female	14 (15.9)	8 (15.7)	6 (16.2)	
Hypertension	60 (68.2)	36 (70.6)	24 (64.9)	0.736
Dyslipidemia	41 (46.6)	26 (51.0)	15 (40.5)	0.452
Diabetes mellitus	24 (27.3)	13 (25.5)	11 (29.7)	0.843
Current smoking	38 (43.2)	24 (47.1)	14 (37.8)	0.520
eGFR, mL/min/1.73 m^2^	75.3 (65.5, 85.4)	73.5 (65.4, 82.7)	76.8 (67.3, 89.2)	0.410
Low-density lipoprotein cholesterol, mg/dL	114 (96, 147)	121 (107, 151)	106 (88, 125)	0.023
High-density lipoprotein cholesterol, mg/dL	45 (40, 56)	44 (40, 54)	47 (41, 59)	0.138
Triglyceride, mg/dL	140 (81, 210)	167 (102, 236)	97 (68, 175)	0.009
Hemoglobin A1C, %	5.9 (5.5, 6.5)	5.9 (5.5, 6.4)	5.9 (5.5, 6.7)	0.830
CRP, mg/dL	0.09 (0.04, 0.29)	0.09 (0.05, 0.29)	0.09 (0.03, 0.29)	0.629
NT-proBNP, pg/mL	199 (107, 493)	226 (141, 703)	181 (85, 345)	0.088
LVEF, %	63 (54, 66)	62 (52, 66)	63 (58, 66)	0.266
Peak CK, U/L	219 (117,463)	246 (156, 570)	230 (106, 544)	0.457
Peak CK-MB, U/L	20 (12, 37)	24 (14, 35)	26 (12, 53)	0.525

Data are presented as number (%), mean ± SD, or median (interquartile range).

CK, creatine kinase; CRP, C-reactive protein; eGFR, estimated glomerular filtration rate; LVEF, left ventricular ejection fraction; NT-proBNP, N-terminal pro-B-type natriuretic peptide.

### Angiographic findings

Angiographic findings are summarized in Table 2. Over half of our study population had a target lesion in the left anterior descending coronary artery (LAD). There was no significant difference in the angiographic findings.

**Table 2:** Angiographic findings.

	All (n = 88)	Layered plaque (n = 51)	Non-layered plaque (n = 37)	P value
Culprit vessel				0.613
LAD	48 (54.5)	30 (58.8)	18 (48.6)	
LCx	18 (20.5)	9 (17.6)	9 (24.3)	
RCA	22 (25.0)	12 (23.5)	10 (27.0)	
QCA analysis				
MLD, mm	0.74 ± 0.38	0.70 ± 0.34	0.79 ± 0.43	0.310
RD, mm	2.75 ± 0.64	2.68 ± 0.55	2.85 ± 0.74	0.237
DS, %	72.7 ± 13.2	73.2 ± 12.8	72.0 ± 14.0	0.695
LL, mm	13.2 ± 5.8	14.0 ± 6.1	12.1 ± 5.2	0.121
TIMI flow grade 0–1	7 (8.0)	4 (8.0)	3 (8.1)	1.000
AHA B2/C	44 (50.6)	35 (68.6)	15 (40.5)	0.337
Multivessel disease	40 (47.6)	27 (55.1)	13 (37.1)	0.161

Data are presented as number (%) or mean ± SD.

DS, diameter stenosis; LAD, left anterior descending coronary artery; LCx, left circumflex coronary artery; LL, lesion length; MLD, minimal lumen diameter; QCA, quantitative coronary angiography; RCA, right coronary artery; RD, reference vessel diameter.

### PCAT findings

**Fig 3:**
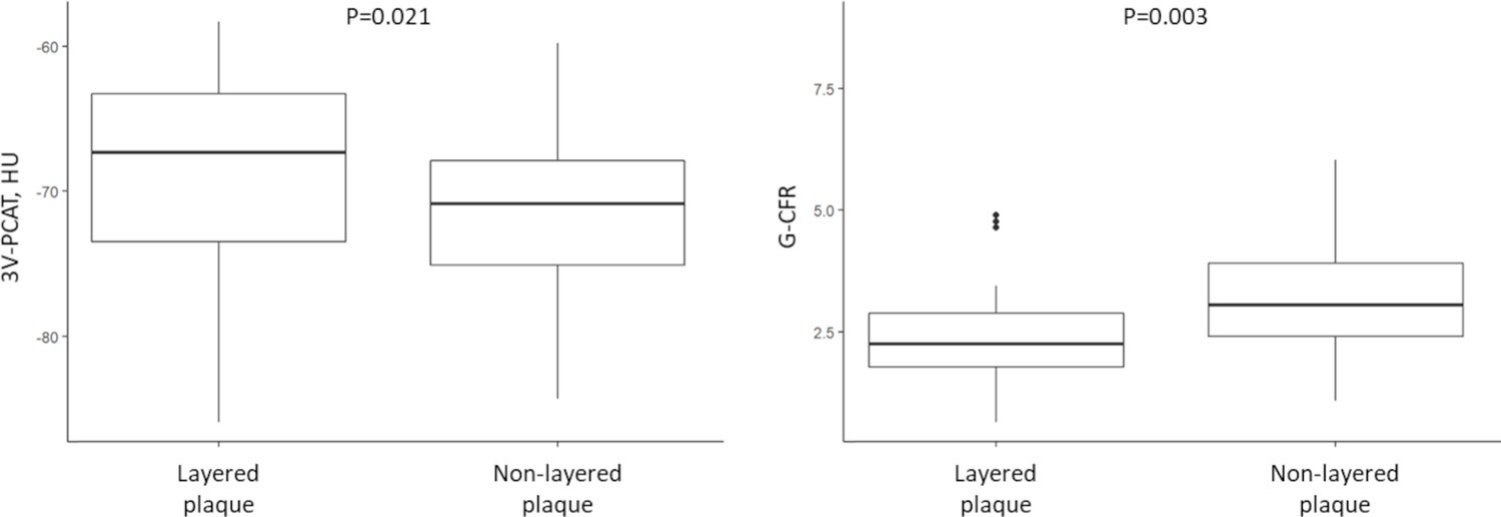
Association of the presence of layered plaque with three-vessel-PCAT attenuation and G-CFR values.

Pre-PCI CCTA-derived PCAT findings are shown in Table 3. The patients with layered plaque had higher three-vessel-PCAT attenuation value (-68.58 ± 6.41 vs. -71.60 ± 5.21 HU, P = 0.021) (Fig 3) and culprit vessel-PCAT attenuation value (-67.69 ± 7.76 vs. -72.07 ± 6.57 HU, P = 0.007) than those with non-layered plaque.

**Table 3:** Pre-PCI CCTA findings.

	All (n = 88)	Layered plaque (n = 51)	Non-layered plaque (n = 37)	P value
PCAT 3-vessel	-69.85 ± 6.09	-68.58 ± 6.41	-71.60 ± 5.21	0.021
PCAT culprit vessel	-69.53 ± 7.57	-67.69 ± 7.76	-72.07 ± 6.57	0.007

Data are presented as mean ± SD.

PCAT, pericoronary adipose tissue.

### OCT findings

Pre-PCI OCT findings are summarized in Table 4. The patients with layered plaque had higher frequency of microvessels than those with non-layered plaque.

**Table 4:** OCT findings.

	All (n = 88)	Layered plaque (n = 51)	Non-layered plaque (n = 37)	P value
Fibroatheroma	75 (85.2)	46 (90.2)	29 (78.4)	0.216
TCFA	42 (47.7)	23 (45.1)	19 (51.4)	0.716
Plaque rupture	48 (54.5)	25 (49.0)	23 (62.2)	0.315
Calcification	37 (42.0)	23 (45.1)	14 (37.8)	0.644
Thrombus	65 (73.9)	37 (72.5)	28 (75.7)	0.933
Microvessel	45 (51.1)	33 (64.7)	12 (32.4)	0.006
Macrophage	74 (84.1)	46 (90.2)	28 (75.7)	0.123
Cholesterol crystal	31 (35.2)	21 (41.2)	10 (27.0)	0.252
MLA, mm^2^	1.15 ± 0.66	1.04 ± 0.60	1.31 ± 0.72	0.057
Thinnest FCT, μm	77 ± 38	84 ± 45	68 ± 23	0.085
Max lipid arc, degree	263.0 ± 66.0	268.2 ± 58.7	258.3 ± 78.4	0.535

Data are presented as number (%) or mean ± SD.

FCT, fibrous cap thickness; MLA, minimal lumen area; TCFA, thin-cap fibroatheroma.

### CMR findings

Post-PCI CMR findings are summarized in Table 5. The patients with layered plaque had lower G-CFR value (median, 2.26 [interquartile range, 1.78, 2.89] vs. 3.06 [2.41, 3.90], P = 0.003) than those with non-layered plaque (Fig 3). CSF and corrected CSF at rest tended to be greater and LV mass was greater in patients with layered plaque. Furthermore, adding the presence of layered plaque to the clinical model (reference model 1) showed significant incremental reclassification ability for predicting low G-CFR (G-CFR < 2.0) (Table 6).

**Table 5:** Post-PCI CMR findings.

	All (n = 88)	Layered plaque (n = 51)	Non-layered plaque (n = 37)	P value
CSF at rest, mL/min	98.71 (77.73, 127.20)	108.20 (87.61, 129.41)	88.91 (63.44, 111.95)	0.013
Corrected CSF at rest, mL/min	112.80 (85.63, 156.22)	128.99 (100.24, 163.52)	94.03 (75.02, 136.56)	0.004
Corrected CSF at rest, mL/min/g	0.86 (0.68, 1.14)	0.94 (0.71, 1.21)	0.82 (0.64, 1.05)	0.171
CSF at hyperemia, mL/min	314.31 (263.40, 360.02)	304.37 (244.60, 356.67)	318.73 (266.97, 363.86)	0.617
CSF at hyperemia, mL/min/g	2.38 (1.65, 3.10)	2.22 (1.52, 3.16)	2.45 (2.08, 2.96)	0.211
G-CFR	2.69 (2.00, 3.38)	2.26 (1.78, 2.89)	3.06 (2.41, 3.90)	0.003
G-CFR <2.0	22 (25.0)	17 (33.3)	5 (13.5)	0.046
EDV, mL	114.0 (96.0, 130.7)	115.0 (96.4, 145.5)	107.6 (93.6, 122.7)	0.230
ESV, mL	42.2 (31.0, 55.5)	42.2 (30.8, 65.5)	41.6 (32.7, 51.9)	0.515
EF, %	61.7 (55.7, 68.2)	61.7 (53.2, 67.7)	59.8 (56.8, 68.4)	0.723
LV mass, g	137.7 (111.3, 154.4)	141.5 (118.7, 157.2)	125.6 (106.4, 144.0)	0.030
LGE volume, mL	1.3 (0.0, 4.6)	1.0 (0.0, 4.3)	2.0 (0.0, 6.6)	0.467
Culprit vessel LGE	33 (37.5)	22 (43.1)	11 (29.7)	0.289
Non-culprit vessel LGE	13 (14.8)	6 (11.8)	7 (18.9)	0.529

Data are presented as number (%) or median (interquartile range).

CSF, coronary sinus flow; EDV, end-diastolic volume; EF, ejection fraction; ESV, end-systolic volume; G-CFR, global coronary flow reserve; LGE, late gadolinium enhancement; LV, left ventricular.

**Table 6:** Prediction model for the presence of G-CFR <2.0.

	C statistics	95% CI	Relative IDI		Continuous NRI	
			value	P value	value	P value
Model 1	0.679	0.550, 0.809	Reference		Reference	
(Age, female, hypertension, diabetes mellitus, dyslipidemia, current smoking, culprit plaque rupture)						
						
Model 2	0.716	0.591, 0.840	0.041	0.056	0.515	0.018
(Model 1 + layered plaque)						

CI, confidence interval; IDI, integrated discrimination improvement; NRI, net reclassification improvement.

## Discussion

The main findings of this study include that the patients with layered plaque at the culprit lesion had higher values of pre-PCI three-vessel-PCAT attenuation and culprit vessel PCAT attenuation and lower values of G-CFR after primary/urgent PCI compared with those with non-layered plaque at the culprit lesion.

### Layered plaque phenotype and coronary inflammation

Several studies have reported the association between coronary atherosclerosis and PCAT attenuation. A recent study demonstrated that PCAT attenuation of culprit lesions of patients with ACS was higher compared with those of non-culprit lesions of ACS patients and highest-grade stenotic lesions of matched control patients [10]. Another recent study showed that PCAT attenuation of the RCA was associated with the localization (culprit/target vs. non-culprit/target vessel), whereas PCAT attenuation of the LAD was associated with the clinical presentation (ACS vs. stable coronary artery disease [CAD]) [8]. Another study reported that patients with plaque rupture showed higher PCAT attenuation in culprit plaque, culprit vessel, and all three coronary arteries compared with those with plaque erosion [21]. Our results extended these studies and demonstrated that the presence of OCT-defined layered plaque at the culprit lesion was associated with higher values of PCAT attenuation of three-vessel and the culprit vessel. An ex vivo validation study for evaluating healed plaque demonstrated that OCT-defined layered structure showed good agreement (93%) with histologically-defined healed plaque [4]. This OCT definition has been frequently used in various previous studies [17, 22–25]. Pathology studies have reported that the presence of healed plaque, represented as OCT-defined layered plaque, suggests previous plaque destabilization and luminal thrombosis with subsequent thrombus organization and debris liberation [1, 2]. Our findings may further highlight the potential link of layered plaque and coronary inflammation of the subtended vessel territory.

### Layered plaque phenotype as a sign of unrecognized myocardial infarction

Healed plaque rupture is represented as the disruption of the fibrous cap that mainly consists of collagen type 1 overlying a necrotic core and superimposed layer of collagen type 3 with looser smooth muscle cell formation [1, 2]. An autopsy study by Burke et al. [2] reported the prevalence of healed plaque was 75% in hearts with acute plaque rupture, 9% in hearts with acute plaque erosion, and 80% in hearts with stable plaque and healed myocardial infarction, indicating a high incidence of subclinical episodes of plaque destabilization and healing in patients with CAD. Healed plaque is likely to be a signature of previous multiple plaque rupture, subclinical luminal thrombosis, distal embolization, and silent myocardial infarction, that may lead to a higher incidence of unrecognized myocardial infarction downstream of the healed plaque [1, 2, 26]. Several studies have suggested an association between the presence of CMR-defined unrecognized myocardial infarction and worse prognosis [27–31]. Recently, the association between OCT-defined healed plaque rupture and worse prognosis has been reported [32]. These studies indicate that the presence of OCT-defined layered plaque at the culprit site is suggestive of unrecognized myocardial infarction of the subtended vessel territory, leading to worse prognosis.

### Layered plaque phenotype and coronary flow reserve

Our results demonstrated the graded difference in G-CFR depending on the presence of OCT-defined layered plaque. An OCT study demonstrated that the presence of TCFA was significantly associated with low pressure-temperature sensor-tipped wire-derived CFR [12]. To the best of our knowledge, the present study is the first report that demonstrated the association between layered plaque phenotype and CMR-derived G-CFR. A recent study reported that CMR-derived G-CFR was an independent predictor of major adverse cardiac events independent of infarct size and conventional risk factors in patients with acute myocardial infarction treated with PCI [18]. CFR is affected by epicardial CAD, diffuse atherosclerosis, vessel remodeling, and microvascular dysfunction on myocardial tissue perfusion [33]. Our findings implied that presence of layered plaque on pre-PCI OCT may indicate the signature of the presence of residual microvascular dysfunction at least partly represented by impaired CFR. Our findings suggest that subclinical thrombosis leading to formation of layered plaque preceding the clinical episode of ACS might affect global myocardial perfusion beyond the myocardial damage of symptomatic ACS resulting in impaired G-CFR, which would link with worse outcomes.

### Clinical implications of this study

Our results explored the clinical significance of layered plaque phenotype on coronary inflammation and coronary flow reserve in patients with NSTE-ACS. Previous OCT studies reported the presence of layered plaque was associated with features of plaque vulnerability [17, 22], plaque progression [23], and worse prognosis [25, 34]. Our findings implied that the detection of layered plaque on pre-PCI OCT may help identify high-risk plaques accompanied by the presence of increased coronary inflammation and impaired coronary flow reserve, which may lead to worse cardiac outcomes. Further studies are warranted to test the hypothesis that aggressive therapeutic intervention targeting the plaque stabilization and prevention of subclinical layered plaque formation may increase coronary flow reserve and improve clinical outcomes.

### Study limitations

First, this is a retrospective single-center observational study and therefore has an intrinsic risk of selection bias. Furthermore, our analysis was performed between the two groups using a small number of patients. Thus, the present study was a merely hypothesis-generating study with a sample size too small to draw definitive conclusions. However, our results suggest that comprehensive assessment by multimodality imaging may provide a novel means of risk stratification in patients with ACS. Second, it is sometimes difficult to differentiate layered plaque phenotype from other plaque components, such as lipid, calcifications, macrophage accumulation, or residual thrombus. However, an extreme caution was exercised to read the underlying structure by the two expert reviewers in this study. Third, the assessment of myocardial segments on CMR was determined by the coronary anatomy by two expert investigators, and no objective method was applied. PC cine-CMR of CS can assess only global myocardial flow but not reginal myocardial flow. Global but not regional myocardial flow assessment using positron emission tomography and its prognostic information has been reported [35]. Fourth, OCT assessment of non-culprit plaque characteristics was not performed. Therefore, the influence of the prevalence of multivessel disease on pericoronary inflammation or the prevalence of layered plaque at the non-culprit vessels was not fully considered. Finally, clinical long-term outcomes were not evaluated. Further studies are warranted to investigate whether the non-invasive CCTA-based assessment of pericoronary inflammation is useful for detection of high-risk features such as OCT-defined layered plaque and low G-CFR, which may lead to worse outcomes.

## Conclusions

The presence of OCT-defined layered plaque at the culprit lesion was associated with increased PCAT inflammation and decreased G-CFR in patients with ACS. OCT assessment of culprit plaque morphology and detection of layered plaque may help identify increased pericoronary inflammation and impaired coronary flow reserve, potentially providing the risk stratification in patients with ACS and residual microvascular dysfunction after PCI.
